# Neuromeningeal tuberculosis in HIV-negative patients: a retrospective study in Rabat, Morocco

**DOI:** 10.11604/pamj.2022.43.31.28669

**Published:** 2022-09-19

**Authors:** Aziz Ahizoune, Amal Satte, Mohamed Ajamat, Amine Raggabi, Abdelhay Lemnouer, Ahmed Bourazza

**Affiliations:** 1 Department of Neurology and Neurophysiology, Mohammed V Military Teaching Hospital, University of King Mohammed V-Souissi, Rabat, Morocco,; 2 Laboratory of Microbacteriology, Mohammed V Military Teaching Hospital, University of King Mohammed V-Souissi, Rabat, Morocco

**Keywords:** *Mycobacterium tuberculosis*, meningitis, HIV-negative patients

## Abstract

Neuro-Meningeal Tuberculosis (NMT) is a severe infection of the central nervous system which causes a public health problem in Morocco and in several countries in the world. In order to describe the epidemiological, clinical, paraclinical and outcome among NMT patients without HIV, we carried out a retrospective study at the neurology department of the Military Hospital of Rabat in Morocco, over a period of 17 years (2000-2017). Forty patients were included with a mean age of 44 years (± 18) and a sex ratio of 1.66. A history evoking the possibility of tuberculous origin was found in 8 patients (20%). Febrile confusion was the most common clinical manifestation and was observed in 22 patients (55%) followed by febrile meningeal syndrome in 12 patients (30%). The main abnormalities noted in brain magnetic resonance imaging (MRI) were: hydrocephalus in 13 cases (32.5%), intra-cranial tuberculomas in 10 patients (25%) and leptomeningitis in 9 cases (22.5%). Cerebrospinal fluid study found clear aspect in 29 patients (75%), direct acid fast bacilli smear examination was positive in 4 patients (10%) and positive culture in 4 patients (10%). The Polymerase chain reaction (PCR) study returned positive in 6 patients (35%) of the 17 patients tested. The outcome was good in 18 patients (45%) while 19 patients suffered from neurological sequelae (47.5%) and 3 cases of death recorded (7.5%). Febrile confusion was the most reported manifestation in our patients. Subacute onset of symptoms was the most predominant feature in our patients as reported in the literature. Our results are consistent with the literature and confirm the severity of this infectious disease, even in HIV-negative patients.

## Introduction

Tuberculosis of the central nervous system (CNS) or neuro-meningeal tuberculosis (NMT) is a serious complication of *Mycobacterium Tuberculosis* (MT) infection that´s causing real public health problems around the world. The diagnosis is often challenging due to the clinical polymorphism and difficulty to isolate the MT. Functional and vital prognosis are at risk in more than 50% of cases despite the availability of efficient antibiotic treatment [1]. The World Health Organization (WHO) has estimated the global incidence of tuberculosis at 10 million new cases with 1.5 million deaths in 2018. In Morocco, the average incidence of tuberculosis is 89 cases/100,000 inhabitants in 2015 [2]. Extrapulmonary forms of tuberculosis are seen in 14% of cases, of which only 1% are neuro-meningeal [1]. Patients who are at risk are mainly children and HIV carriers, in whom the evolution can be rapidly fatal [1]. Early diagnosis of tuberculous meningitis remains a great challenge as symptoms such as fever, headache, vomiting, etc., are not specific. The identification of acid-fast bacilli in the cerebrospinal fluid (CSF) and the culture of MT are specific for the diagnosis, but they lack sensitivity [3]. Most often, the diagnosis of tuberculous meningitis is based on clinical suspicion combined with empirical decision-making [3]. The study of immunocompetent patients with this neuro-meningeal infection is rarely reported. The objective of this study is to identify the epidemiological, clinical, paraclinical, and evolutionary characteristics of NMT in this population category.

## Methods

**Study design and setting:** we conducted a retrospective study of patients with a diagnosis of NMT with HIV-negative serology. This study was conducted over a period of 17 years, from January 2000 to September 2017, and took place at the Neurology Department of the Mohammed V Military Teaching Hospital in Rabat. This hospital covers a large region of Morocco. All patients diagnosed with NMT and who were HIV seronegative were retrospectively enrolled in this study. We reviewed the medical records of patients admitted to the neurology department. Outcome and survival were assessed from the follow-up medical records.

**Study population:** we included medical records of patients aged more than 18 years old with NMT and tested negative for HIV. The diagnosis of NMT was based on clinical, biological and radiological characteristics and response to antibacillary treatments. Patients underwent a complete neurological and somatic examination, a lumbar puncture, brain imaging (CT scan and/or brain MRI), standard chest X-ray and blood samples. Spinal cord imaging (CT scan or MRI) was done in patients with manifestations of spinal cord disorders. Cerebrospinal fluid (CSF) study was based on cytobacteriological and biochemical examination (pleocytosis if white blood cells > 5 elements/mm^3^, hyperproteinorachia > 45 mg/dl, hypoglycorachia if < 50% of concomitant blood glucose). CSF culture was performed on Lowenstein Jensen medium and bacterial genome was searched by PCR technique. All patients were tested for MT in sputum, urine and gastric tubing. The biopsy was performed when there was no bacteriological evidence with the presence of a radiological mass syndrome. Once the diagnosis was made, antibacillary treatment was started in the hospital department and patient follow-up was present on all patients' records for a minimum of 12 months after discharge. The treatment regimen followed WHO recommendations and was based on a quadruple therapy combining isoniazid, rifampicin, pyrazinamide and ethambutol (RHZE) for two months, followed by dual therapy (RH: rifampicin and isoniazid) for seven to ten months. Short-term corticosteroid therapy for 1 month and ½ was administered in cases of significant hyperproteinorachia or neurological signs of focalization. Treatment efficacy was judged primarily on clinical improvement and normalization of biological and radiological parameters. Patients with unknown HIV status, and who had unreadable or empty files, and those discharged against medical advice were excluded.

**Data collection:** in this work, patient data were collected from their medical records. Age, sex, clinical data, CSF study, neuroimaging results, biological, microbiological and anatomopathological tests, management, length of stay and follow-up of the included patients were specified.

**Statistical methods:** data were analysed using SPSS Statistics version 18 for Windows (Chicago, Illinois, USA). Categorical data were summarised by frequencies and percentages, while numerical data were summarised by medians and interquartile ranges, or means and standard deviations according to the normal or abnormal distribution of the variables.

**Ethical consideration:** this study was approved by the Ethics Committee of the Faculty of Medicine and Biomedical Sciences. Data were collected anonymously from medical records.

## Results

**General characteristics:** forty patients were included with a mean age of 44 years (±18) and extremes ranging from 19 to 84 years. The sex ratio was 1.66 in favour of frequent male involvement. At the anamnesis, we found a context pointing to a tuberculosis origin in 8 patients (20%); a notion of tuberculosis contagion in 5 patients (12.5%) and 3 cases had a history of treated pulmonary tuberculosis (7.5%). The other main antecedents found in our study: rheumatic disease under oral corticosteroid therapy in 3 patients (7.5%), diabetes in 3 patients (7.5%), asthma in 2 patients (5%), active smoking in 3 patients (7.5%), and surgery for lumbar disc herniation in less than 12 months in 2 patients (5%). The median time to hospital admission after symptom onset was 15 days.

**Clinical and paraclinical presentations:** the clinical manifestations at admission and radiological findings are summarized in Table 1, Table 2 respectively. Lumbar puncture was performed in 39 patients, while the other patient had a pseudotumoral syndrome on cerebral MRI, hence the use of stereotactic biopsy which objectified the presence of lesions specific to tubercular origin. The results of the CSF study and the radiological abnormalities found on the standard chest X-ray are summarized in Table 3. Identification of MT was possible in 11 patients: 8 patients (20%) at CSF level and 3 patients (7.5%) at other sites: cytobacteriological examination of sputum in one patient, an anatomopathological study of a pseudotumoral process of the pontocerebellar angle in a 2nd case and cervical adenopathy in a 3^rd^ case.

**Table 1:** clinical features on admission of patients with neuromeningeal tuberculosis (N=40)

Variable	Frequency	Percent
**Febrile meningeal syndrome**	6	15
**Febrile meningeal syndrome associated to**	6	15
Sphincter disorders	2	5
Cranial nerve palsy (3^rd^ nerve)	1	2.5
Hemiparesis	1	2.5
Cerebellar ataxia	2	5
Seizure	3	7.5
**Febrile confusion with**	22	55
Areflexic paraparesis	8	20
Sphincter disorders	5	12.5
Cranial nerve palsy	10	25
*7^th^ nerve*	5	12.5
*3^rd^ nerve*	3	7.5
*6^th^ nerve*	4	10
Hemiparesis	2	5
Cerebellar ataxia	4	10
Seizure	2	5
**Psychiatric manifestations**	2	5
Psychotic attack	1	2.5
Motor agitation	1	2.5
**Myelopathy**	2	5
**Increased intracranial pressure** syndrome with involvement of 7^th^ and 8^th^ cranial nerves	1	2.5
**Impaired consciousness with fever**	1	2.5

N=Total number of patients

**Table 2:** results of cerebro-medullary neuroimaging of patients with neuro-meningeal tuberculosis (N=40)

	Frequency	Percent
**Abnormal Brain CT scan**	18	45
Hydrocephalus	13	32.5
Cerebral edema	4	10
Leptomeningitis	5	12.5
Cerebellar abscess	1	2.5
Thalamic stroke	1	2.5
**Abnormal Brain MRI**	25	62.5
Hydrocephalus	13	32.5
Cerebral Tuberculomas	9	22.5
Leptomeningitis	9	22.5
Abscess	2	5
**Cerebral involvement**	1	2.5
**Cerebellar involvement**	2	5
Fronto-parietal subdural empyema	1	2.5
Tuberculoma of the cerebellopontine angle	1	2.5
Ischemic stroke:	5	12.5
**Thalamic**	1	2.5
**Capsulolenticular**	3	7.5
**Head of the caudate nucleus**	1	2.5
Encephalitis	3	7.5
**Abnormal Spinal MRI**	8	20
Arachnoiditis	6	15
Spondylodiscitis	4	10
Tuberculoma of the medullary cone	1	2.5
Subdural abscess of the dorsal spine with arachnoiditis	1	2.5
Abscess of psoas muscle	2	5

N=Total number of patients

**Table 3:** cytochemical and bacteriological data of cerebrospinal fluid with standard chest X-ray aspects

Study of CSF (N1= 39)	Frequency	Percent
**Appearance**		
Clear	29	75
Turbid	6	15
Xanthochromic	4	10
**Chemistry:**		
Elevated levels of Protein	39	100
Low glucose	35	90
**Cells:**		
Predominance of lymphocytes	30	77
Predominance of neutrophils	5	13
Mixed	4	10
**Bacteriology:**		
Direct examination: presence of Acid-Fast Bacilli	4	10
Positive mycobacterial culture	4	10
**Positive PCR in CSF (N2= 17 cases)**	6	35
**Abnormal Standard chest X-ray: (N3=40(100%))**	15	37.5
Interstitial syndrome	5	12.5
Pulmonary military	8	20
Apical opacity with pleurisy	4	10
Mediastinal adenopathies	4	10
Apical pulmonary nodules	1	2.5

**CSF:** cerebrospinal fluid, **N1:** the number of patients in which a lumbar puncture was performed. In the other patient, the CSF study was impossible because of intracranial hypertension syndrome. **N2:** number of patients tested with PCR. **N3:** total number of patients

**Management and outcome:** all patients received antibacillary treatment. Oral corticosteroid therapy was added in 32 patients (80%). Ten patients (25%) underwent ventriculo-peritoneal derivation. The outcome was favourable in 18 patients (45%) and fatal in 3 patients (7.5%). Thirteen patients (32.5%) presented motor sequelae such as paraparesis or hemiparesis. The cerebellar syndrome was persistent in 5 patients (12.5%) and 7 patients (17.5%) had developed epilepsy.

## Discussion

The clinical and paraclinical polymorphism of NMT is accompanied by a diagnostic delay in patients with late initiation of antibacillary therapy. The diagnosis of NMT in our patients was made on the basis of epidemiological, clinical, CSF, radiological criteria, and evidence of tuberculosis elsewhere. It has been reported that NMT patients co-infected with HIV were more at risk of poor treatment outcomes and death [3]. In this work, we aimed to examine the epidemiological, clinical, paraclinical data, and the outcome of patients with NMT who had HIV-negative status in Morocco. Our study found a predominance of this infection in males and a subacute character of NMT as reported in the literature [3]. The presence of an antecedent pointing to a tubercular origin is reported in 21.6% of cases and should always be looked for, as it helps to direct paraclinical investigations and early treatment [3]. Febrile confusion was the most reported manifestation in our patients, whereas in the study conducted by Zhang *et al*. in 2015 on 401 HIV-negative NMT patients; the confusion was seen in 25.9% of cases [4]. Our result could be explained by the diagnostic delay and wandering that are often encountered during this cerebro-meningeal infection. The meningeal syndrome is the most described manifestation in NMT without taking into account the HIV serological status [5]. In the population with negative HIV serology we found that the meningeal syndrome is described in 24.9% of patients as approximately what was seen in our patients [4].

According to recent data, the cranial nerves are affected in 25% of cases in NMT, and the external oculomotor nerve is the most affected, whereas in our study we noted that the facial nerve was the most affected [6]. Clinical presentations of myelopathy are seen in 10% of cases and may or may not be associated with cerebro-meningeal lesions [7]. This presentation was found in only 2 patients (5%) where spinal cord MRI had a primary role in the etiologic diagnosis by showing characteristic aspects of the tubercular origin. Two patients (5%) initially presented with psychiatric manifestations who had been diagnosed with NMT, this finding is rarely reported in the literature [8]. Seizures are seen in 18.5% of cases in NMT, regardless of HIV status [3]. For the study published by Zhang *et al*. in 2015: epileptic seizures and consciousness impairment are present in 6% and 10% of cases, respectively, in HIV-negative patients [4]. For our study; we have an intermediate proportion that manifested seizures on admission, while impairment of consciousness was seen in only one patient (2.5%) on admission.

Our study matches with literature regarding the superiority of brain MRI over computed tomography (CT) scan by showing abnormalities suggesting NMT in 74% of cases. Leptomeningitis is the most reported abnormality in several series with a proportion of 43.3%, while in our patients we found hydrocephalus which is at the first level followed by leptomeningitis and cerebral tuberculomas. [5] Leptomeningitis is easily detected by brain MRI and is more frequent in HIV-positive patients compared to HIV-negative patients [9]. According to the literature, there is no difference between CT and brain MRI for the detection of hydrocephalus, which is consistent with our study findings [10]. Ischemic strokes in NMT context are reported with a rate of 22.7% [5]. The stroke during NMT is mainly located in a cerebral zone called “tubercular zone” which includes the head of the caudate nucleus, the antero-medial part of the thalamus, and the capsulo-lenticular region [11]. Concerning our study, the site of strokes had the same topography and this was explained by the fragility of this area to ischemia during NMT. The incidence of tuberculoma and strokes during NMT is similar between HIV-negative and HIV-positive patients while tuberculous abscesses are frequently reported in HIV-positive patients [12]. Cerebral tuberculomas may or may not be associated with meningitis and are reported in 22.4% of cases [3]. Our study is consistent with what has been reported and one of our patients had a tuberculoma in the posterior cerebral fossa whose etiological diagnosis was made by stereotactic biopsy. Concomitant lung involvement in NMT was frequently reported in the literature with a proportion of 53.2%, which is close to our findings [5]. Standard chest X-rays are of great diagnostic value because they can suggest the diagnosis when they show characteristic lesions of pulmonary tuberculosis [13].

The CSF aspect is classically clear in 80-90% of cases with a predominantly lymphocytic pleocytosis (30-90%) compared to neutrophils (10-70%) and this is consistent with our results [14]. Hyperproteinorachia and hypoglycorachia are common in NMT as found in our study. In HIV-positive patients, the same results are found except for the number of leukocytes, which is lower [14]. According to the literature; MT was identified in CSF by direct bacteriological examination and culture in 10% and 23.8% of cases, respectively [3]. PCR was positive in 22.3% of cases, corresponding to the same findings in our study [3]. The low sensitivity of these tests explains the diagnostic difficulties often encountered during NMT and thus the delays in initiating anti-tuberculosis treatment. Thwaites *et al*. conducted a comparative study in 2005 on the characteristics of CSF between HIV-negative and HIV-positive patients and found no difference between these groups except for the isolation of MT in the CSF which was superior in HIV-positive patients [15].

The prognosis of NMT is bleak as it continues to cause death and neurological sequelae, even in HIV-negative patients. In 2018, the WHO reported that tuberculosis caused 1.2 million deaths in HIV-negative patients and 251,000 deaths in HIV-positive patients [15]. Our results are consistent with the literature and affirm the severity of this infectious disease, even in HIV-negative patients. Short-term corticosteroid therapy plays an important role as adjunctive therapy to anti-bacillary drugs because it prolongs the survival of HIV-negative patients with NMT [16].

Our work is very interesting because it allows us to assess the variability that can be observed in NMT among immunocompetent patients, both clinically and paraclinically. The limitations of this work are, on the one hand, that it is a retrospective and single-centered study on a small sample and, on the other hand, that it would be very interesting if it were a comparative study with HIV-positive patients affected by NMT.

## Conclusion

Neuromeningeal tuberculosis is a serious and polymorphic disease that always causes health and socio-economic problems. Febrile confusion was the most reported manifestation in our patients, followed by the febrile meningeal syndrome. Subacute onset of symptoms was the most predominant feature in our patients as reported in the literature. Given the low sensitivity of tests to identify MT, careful clinical and paraclinical evaluation is required to confirm the tuberculous nature of the neuromeningeal involvement. Our results confirm the severity of this infectious disease, even in HIV-negative patients.

### What is known about this topic

- Neuromeningeal tuberculosis is a public health problem that is often difficult and late to diagnose;
- Clinical and paraclinical presentations are varied;
- Functional and vital prognosis are at stake.

### What this study adds

- Epidemiological, clinical, and para-clinical data help to guide the etiologic investigation;
- Complications and sequelae can be seen even in HIV-negative people;
- We added 40 cases of neuromeningeal tuberculosis in non-HIV patients.
